# Arbitrary Font Generation by Encoder Learning of Disentangled Features

**DOI:** 10.3390/s22062374

**Published:** 2022-03-19

**Authors:** Jeong-Sik Lee, Rock-Hyun Baek, Hyun-Chul Choi

**Affiliations:** 1 ICVSLab., Department of Electronic Engineering, Yeungnam University, 280 Daehak-ro, Gyeongsan 38541, Gyeongbuk, Korea; a2819z@ynu.ac.kr; 2 Department of Electrical Engineering, Pohang University of Science and Technology, Pohang 37673, Gyeongbuk, Korea; rh.baek@postech.ac.kr

**Keywords:** arbitrary font generation, feature disentanglement, stacked input, consistency loss, hallucinated input

## Abstract

Making a new font requires graphical designs for all base characters, and this designing process consumes lots of time and human resources. Especially for languages including a large number of combinations of consonants and vowels, it is a heavy burden to design all such combinations independently. Automatic font generation methods have been proposed to reduce this labor-intensive design problem. Most of the methods are GAN-based approaches, and they are limited to generate the trained fonts. In some previous methods, they used two encoders, one for content, the other for style, but their disentanglement of content and style is not sufficiently effective in generating arbitrary fonts. Arbitrary font generation is a challenging task because learning text and font design separately from given font images is very difficult, where the font images have both text content and font style in each image. In this paper, we propose a new automatic font generation method to solve this disentanglement problem. First, we use two stacked inputs, i.e., images with the same text but different font style as content input and images with the same font style but different text as style input. Second, we propose new consistency losses that force any combination of encoded features of the stacked inputs to have the same values. In our experiments, we proved that our method can extract consistent features of text contents and font styles by separating content and style encoders and this works well for generating unseen font design from a small number of reference font images that are human-designed. Comparing to the previous methods, the font designs generated with our method showed better quality both qualitatively and quantitatively than those with the previous methods for Korean, Chinese, and English characters. e.g., 17.84 lower FID in unseen font compared to other methods.

## 1. Introduction

**Figure 1:**
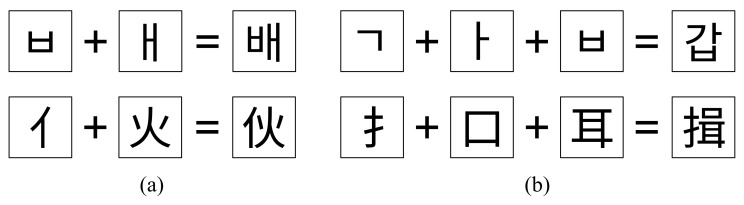
Example of combination characters. (**a**) example of a combination of two components, (**b**) example of a combination of three components. Hangul has 11,172 syllables in a combination of 2 or 3 components, and there are over 50,000 combinations in Chinese characters.

For the combination characters such as Hangul, the Korean characters with 11,172 characters, including all combinations of consonants and vowels, and Chinese characters with enormous combinations of hieroglyphs, as shown in Figure 1, font design by human designers is labor-intensive and requires a huge time to deal with much greater number of characters compared to English alphabet font design, which requires only 52 letters including upper and lower case letters. To solve this inefficiency in the font design process, automatic font generation methods using deep learning [1,2,3,4,5] have recently been proposed.

These font generation methods [1,2,3,4,5] showed output font images of high quality by using an Image-to-Image translation technique based on Generative Adversarial Network (GAN) [6]. However, there are problems of training instability in these GAN-based approaches. Various GAN losses [7,8,9] and training techniques [10,11] have been proposed to solve the mode collapsing problem, but it is not fully solved yet, and there is another problem of deciding when to stop training. In addition, GAN-based methods need to train new networks to generate new unseen fonts. To generate arbitrary fonts, new network architectures [5,12] have been proposed where two encoders were used to extract text content and font style features, respectively, from different font images, and the features were mixed to be decoded into a new font image. However, for handwriting fonts, it is still difficult to generate font images using an input image of a font style reference that has quick strokes like handwritten characters and to make consistent font images regardless of text content reference in another font style. These difficulties come from any input image of text content or font style having both text content and font style in itself and that those two features are not easily disentangled.

In this work, we propose a new approach for automatic font generation to overcome those existing problems in the previous methods. Instead of GAN with training instability, we use an encoder–transformer–decoder architecture based on neural-style transfer and two separate encoders, one for extracting text content and the other for font style. For training two separate encoders to extract disentangled text content and font style features, we propose to use stacked inputs for both text content, i.e., images with the same character in different font styles, and font style, i.e., images with different characters in the same font style. Using these stacked inputs alone does not guarantee that the encoders extract completely disentangled text content and the font style features. So, we additionally use a new loss called consistency loss that enforces any combination of encoded features of the stacked inputs to have the same value, and this allows the encoder to extract consistently disentangled features of the stacked images for text content and font style. These disentangled features enable consistently high quality of font representation in arbitrary font generation. Although we need multiple images for training with stacked input and consistency loss, in the test phase, we show that our method can generate arbitrary fonts with only two input images for text content and font style, respectively. Our contributions are summarized as follows:

- We disentangle text content features and font style features from font images by training two encoders using stacked inputs and consistency loss.
- We propose an end-to-end learning and inference method to generate arbitrary fonts from a pair of stacked inputs or from a pair of character content and font style images.
- We show that a learnable transformer layer with ResBlock is compatible with our method but is not recommended for a qualified arbitrary font generation
- Our method is compatible with the previously used style interpolation technique and can generate intermediate font styles between several different font styles.

In the remaining parts of this paper, we will firstly explain the previous study related to our work in Section 2; secondly, we will explain the details of our method in Section 3; thirdly, we will experimentally verify its effectiveness in Section 4; and finally, we will conclude this work.

## 2. Related Works

### 2.1. Font Generation

Most of the previous font generation methods are based on Image-to-Image Translation by using GAN. zi2zi [1] used one-hot encoding for font styles. The one-hot encoded feature is a fixed-length vector that has a single non-zero value corresponding to a specific desired font style and zero values for the other font styles. This method is very efficient for font style representation in latent space, but it is impossible to generate unseen fonts using this method because one-hot encoding cannot embed unseen font styles in its fixed-length vector. Li et al. [5] modified FUNIT [13] to enable font effect transfer and proposed a few-shot training method to generate unseen font images. However, since GAN-based networks need re-training or fine-tuning to generate unseen font styles, it is difficult to apply those methods for arbitrary font generation. In addition, there is training instability in GAN, i.e., mode collapse or training oscillation, and many works [10,14] have been conducted to solve these problems, but the problem still exists.

Zhang et al. [12], instead of using GAN, proposed to use a network architecture with two encoders, one for content and the other for style. They generated arbitrary fonts by mixing the content and style features of input images followed by decoding the mixed feature. However, their method cannot generate fonts with thin strokes because their encoders did not effectively disentangle the extracted text content and font style from an image [15].

W-Net [16] and OCFGNet [17] trained content and style encoders by using classifiers for the content and style encoders to output text content feature and font style feature corresponding to input character and font images, respectively. Their classifiers output the categories of the features from the encoders. The difference between the classifier output and ground truth, called categorical loss, is used for encoder training. However, a classifier with a limited number of categories is not applicable to arbitrary content and style encoding. In addition, they simply concatenated content and style features and decoded the concatenated features, but a simple concatenation of two features is known for its ineffectiveness in arbitrary style transfer [18].

SA-VAE [19] used a human-defined content encoder according to the results of input text content recognition to cover the complicated structure of Chinese characters. This method trained a style encoder for different characters in a same font style to have a same style representation by using KL-divergence loss. However, SA-VAE requires designing human-defined codes for a large number of Chinese characters.

Some studies [15,20,21] did not encode the entire image to extract style information but, instead, extracted style information for each component that makes up a syllable and used it to generate fonts. However, this requires additional labels for components and is not suitable for such languages with a large number of components such as Chinese.

We summarized the mentioned works in Table 1.

### 2.2. Image-to-Image Translation

Image-to-Image Translation [22,23,24,25] has been studied to learn a mapping between two different domains and applied to style transfer such as image coloring, day/night changes, and map generation. These methods require heavy laboring in building a pairwise dataset for network training. CycleGAN [23] was proposed to relieve this requirement of a pairwise dataset in the training phase by using cycle consistency loss. Since this loss considers only the differences between the original data and the reconstructed data through cyclic paths, i.e., source–target–source or target–source–target, unpaired data can be used in loss calculation. However, a huge amount of data are still necessary to map from a source domain to a target domain, and this is not suitable in a real-world situation where a very small amount of sample data are available. FUNIT [13] has recently been studied and is capable of image translation with a few samples. In addition, mapping to unseen domains is possible with this method.

### 2.3. Style Transfer

As the first neural-network-based style transferring method, Gatys et al. [26] proposed a gradient-based optimization technique. They generated a stylized image from a noise image by iteratively updating each pixel value to minimize the content and style differences between the output image and the input content/style images. However, this is a very time-consuming method, and for real-time style transfer, Johnson et al. [27] introduced the use of a feed-forward network instead of iterative optimization.

Huang et al. [18] proposed an AdaIN layer to enable arbitrary style transfer. They extracted features of input content and style images using a pre-trained VGG [28] network and matched the mean and standard deviation of the content feature to those of the style feature by using an AdaIN layer. Sheng et al. [29] used a patch-based style decorator that considers not only the overall style of the style image but also the local style of the style image. This decorator performed better in transferring detailed style than AdaIN layer, which considers only overall style with mean and standard deviation.

However, those methods are using one pre-train encoder network for both content and style extraction without disentangling them.

## 3. Method

In this section, firstly, we introduce our network architecture for arbitrary font generation. Secondly, we explain stacked input and consistency loss for disentangling text content and font style.

### 3.1. Network Structure

**Figure 2:**
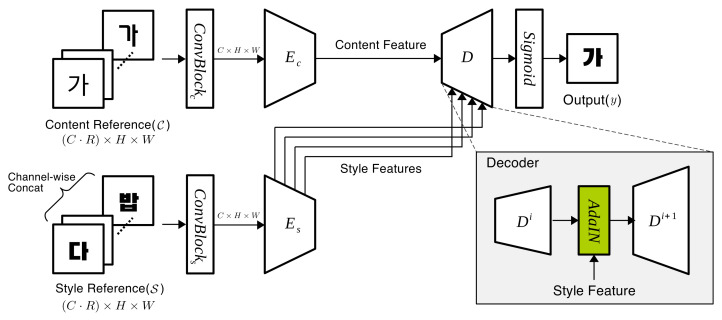
An overview of our proposed method with a few reference images. Images of content reference (C) share the same content, but style and images of style reference (S) share the same style but content.

As shown in Figure 2, our network consisted of a content encoder $E_{c}$, style encoder $E_{s}$, decoder *D*, and several AdaIN layers between decoder layers. The encoders used VGG-19 [28] feature extractor up to conv4_1 and the decoder had the encoder’s mirrored structure. At the end of the decoder, we used sigmoid modules to limit the output pixel values into the rage of [0, 1]. The content encoder extracted content feature map *x* only from its conv4_1 layer, and the style encoder extracted style feature maps, $y_{i}$ for i=1,…,4, from its (conv1_4, conv3_1, conv2_1, conv1_1) layers. These features extracted by two encoders passed through the decoder. In this decoding process, as the multi-level style transfer of Avatar-net [29], the content feature map *x* was transformed by *i*-th style feature map $y_{i}$ in the *i*-th AdaIN layer, where the mean and standard deviation of content feature were changed into those of the style feature using Equation (1), as shown in Figure 2:

(1) $$\mathrm{AdaIN}\left(x,y_{i}\right)=\sigma \left(y_{i}\right)\left(\frac{x-\mu (x)}{\sigma (x)}\right)+\mu \left(y_{i}\right)$$

where σ(x) is the standard deviation of *x*, and μ(x) is mean of *x*.

### 3.2. Stacked Input and Consistency Loss

Some previous methods [12,13] used two encoders, one for content and the other for style, for generating unseen fonts. However, it is difficult to extract disentangled content and style features by simply using those two separate encoders because the pre-trained encoders are just optimized to extract semantic features from a font image, which includes both text content and font style in a feature map. If disentangling fails, font generation using content reference images with the same character but in different font styles may result in different font images because the font style of the reference images affects the content feature of the images. Similarly, poor font generation results may appear when images in the same font style but with different characters are used as a style reference. To avoid this inconsistent and poor result in font generation, we propose using both stacked input and consistency loss for the encoders to extract disentangled text content and font style features.

We define stacked input as a set of images of the same size. As shown in Figure 2, an image set $C=\{c_{1},c_{2},\ldots ,c_{R}\}$ with the same character in different font styles was used as content reference input and another image set $S=\{s_{1},s_{2},\ldots ,s_{R}\}$ with different characters in the same font style as style reference input. Here, one convolution layer, ${ConvBlock}_{c}$ or ${ConvBlock}_{s}$, was added in front of each encoder. The channels of the stacked input were compressed from C×R into *C*, maintaining the spatial size H×W through this convolution layer, and become compatible with the VGG-19 encoder of 3-channel input.

If the encoders extract common features from the stacked inputs, then the extracted features represent text content of content reference input and font style of style reference input since the images of content reference have the same character, and the images of style reference have the same font style.

**Figure 3:**
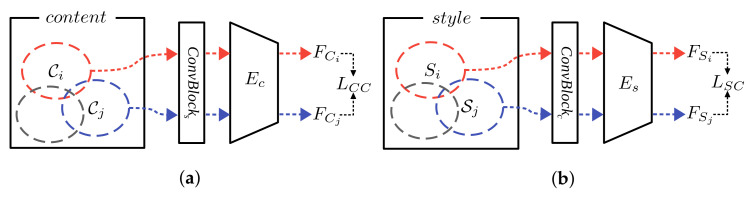
Concept of feature consistency loss: (**a**) Content reference, images with the same character in different font styles; (**b**) Style reference, images with different characters in the same font style. Different colors dotted circles mean sets that have the same content (or style) but different style (or content).

However, disentangled feature extraction is not guaranteed by using stacked input alone because there is no constraint to enforce the extracted features to represent common features of the stacked input. Therefore, we used a new loss called consistency loss to enforce common feature extraction from the stacked input. The consistency loss is calculated through the following process, as shown in Figure 3. Assuming *R* images are in a stacked input, two content stacked inputs, $C_{i}$ and $C_{j}$, were obtained by randomly sampling *R* images twice from a training set of content images (Figure 3a), which have the same text contents in different font styles. In the same manner, two style stacked inputs, $S_{i}$ and $S_{j}$, were obtained by randomly sampling *R* images twice from the training set of style images (Figure 3b), which have different text contents in the same font style. Then, the stacked inputs went through the encoders, and the extracted features of the stacked inputs ($F_{C_{i}}$, $F_{C_{j}}$, $F_{S_{i}}$, and $F_{S_{j}}$) were used to calculate the content consistency loss $L_{CC}$ as Equation (2) and the style consistency loss $L_{SC}$ as Equation (3):

(2) $$L_{CC}={∥F_{C_{i}}-F_{C_{j}}∥}_{2}^{2}$$

(3) $$L_{SC}={∥F_{S_{i}}-F_{S_{j}}∥}_{2}^{2}$$

Finally, the consistency losses $L_{consistency}$ were calculated as their summation (Equation (4)):

(4) $$L_{consistency}=L_{CC}+L_{SC}$$

Using this consistency loss forced the extracted features from the same encoder to have the same value in training phase.

In the training phase, the stacked inputs and consistency loss worked for learning two common feature extractors, one for text content and the other for font style. The stacked inputs were also used in the testing phase to obtain the disentangled content or style features as in the training phase, but it is not easy to arrange and input multiple images in a real situation. To relieve the requirement of stacked input in the test phase, we can use hallucinated input, i.e., a set of the duplicated image from a single image, since the encoders are already trained to extract disentangled features. We call the original stacked input the *real stack* and the hallucinated input the *hallucinated stack*.

### 3.3. Training Font Style Transfer

In addition to our consistency loss, additional losses are needed to train an entire network for font style transfer, i.e., generating font image of given character and font style. We adopted the pixel reconstruction loss $L_{pixel}$ and the perceptual loss $L_{perceptual}$ from [27]. $L_{pixel}$ is defined as the L1 distance between the generated font image (*y*) and the ground truth ($\hat{y}$) in pixel level (Equation (5)) and forces the network to generate a font image from an encoded feature. $L_{perceptual}$ is defined as the L2 distance between two encoded features of the generated font image (*y*) and the ground truth ($\hat{y}$) extracted at conv1_1, conv2_1, conv3_1, and conv4_1 layers of pre-trained VGG-19 with ImageNet [30] dataset (Equation (6)) and forces the generated font image to have the same perceptual feature of the ground truth image:

(5) $$L_{pixel}={∥y-\hat{y}∥}_{1}$$

(6) $$L_{perceptual}=\sum_{i}^{N}{∥VGG_{i}\left(y\right)-VGG_{i}\left(\hat{y}\right)∥}_{2}^{2}$$

Other pre-trained networks might be used to calculate the perceptual loss, but we used VGG-19 since the encoder in the proposed method follows the VGG structure.

Finally, the total training loss for to end-to-end learning of our font generation network was calculated as the weighted summation of $L_{pixel}$, $L_{perceptual}$, and $L_{consistency}$ (Equation (7)):

(7) $$L_{total}=\alpha L_{pixel}+\beta L_{perceptual}+\gamma L_{consistency}$$

where α,β,γ are the weight of each loss function. We use α=10,β=0.01,γ=0.01, which show the best performance qualitatively and quantitatively, for all experiments except the ablation study.

## 4. Experiments

In this section, we will experimentally analyze the benefits of using our stacked input and consistency loss in arbitrary font generation by conducting ablation studies for each component of our method and by comparing result images of the previous methods with ours.

### 4.1. Experimental Setup

For our experiments, we collected 278 copyleft Korean fonts released on the website of Noonnu [31], 200 Chinese fonts, and 200 English fonts from the Internet [32,33]. Then, we randomly divided the Korean font set into a training font set of 238 fonts and a test font set of 40 fonts without overlapping and divided the Chinese and English font sets into a training font set of 185 fonts and a test font set of 15 fonts, respectively. We summarized the split ratio between train and test datasets for each language in Table 2. For network training, we made a training dataset by making 2048 character images of 64×64 pixel size for each font in the training font set of Korean characters. To evaluate the performance of arbitrary font generation, we made a test dataset by making 302 character images of 64×64 pixel size for each font in the test font set of Korean characters. For Chinese characters, we made 3000 character images for training and around 300 character images for test in the same size specification of Korean characters. For English, we used all alphabet characters for both training and testing. For training parameters, we used Adam [34] optimizer with a batch size of 32 and a learning rate of $10^{-4}$. All experiments were conducted with Pytorch v1.6.0 framework, CUDA v11.1, and CuDNN v8.0.2 on a single NVIDIA GTX 1080 Ti device.

### 4.2. Effect of Stacked Input and Consistency Loss

**Figure 4:**
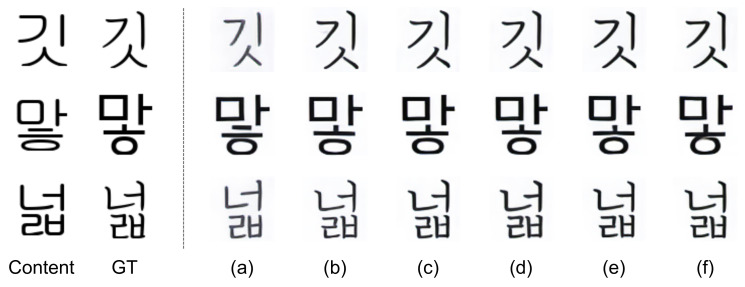
Examples of the generated images for unseen font: Content: content image, GT: image of ground truth font, (**a**–**f**): generated images with (**a**) single input, (**b**) 4 stack, (**c**) 8 stack, (**d**) 16 stack, (**e**) 24 stack, and (**f**) 32 stack for both content and style inputs.

To show how well our stacked input and consistency loss work for feature disentanglement, we analyzed the performance of using stacked input and consistency loss for the varying number of stacked images. Figure 4 shows some samples of generated font images from unseen font style references for the varying number of images in stacked input. Here, the results of the single input (Figure 4a) were obtained by using only one image (labeled as ‘Content’ in Figure 4) as the content reference input, and the other results (Figure 4b–f) were obtained by using multiple images with the same text content in different font styles as the stacked content reference input.

The generated font images with single input (Figure 4a) look much different from the ground truth images (Figure 4 GT) while the results with stacked input (Figure 4b–f) are very close to the ground truth images in font style. By using reference inputs with a single image, encoders were not trained to extract disentangled features of content and style respectively. And, therefore, these entangled features are mixed in AdaIN layers and decoded into poorly generated font images. In contrast, by using our stacked input and consistency loss, encoders were trained to extract the common feature of each stacked input. This led to disentangled features and resulted in desired font generation.

To quantitatively evaluate the font generation performance of our stacked input and consistency loss, we calculated Frechet Inception Distance (FID) [10], a popular measurement for generative model evaluation, between the generated font images and the ground truth images for the training dataset and testing dataset in Section 4.1. FID measures the distance between two distributions of the generated font images and the ground truth images.

**Figure 5:**
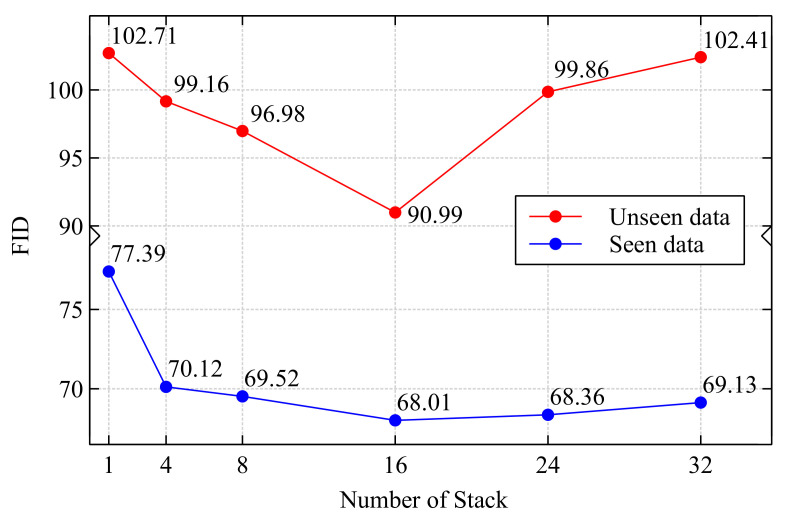
FID vs. number of stacked images.

Figure 5 shows FID according to the number of stacked images. As the number of stack increases, FID gradually decreases as the number of stacks increases for both seen font (blue line on Figure 5) and unseen font (red line on Figure 5). However, FID starts to increase when the number of images in a stack is larger than 16. This increasing FID of the unseen font seems to be caused by overfitting. As the number of images in a stack (*R*) increases, the number of parameters in the convolution layers which are attached to the front of the encoders (${ConvBlock}_{c}$ and ${ConvBlock}_{s}$) in Figure 2 also increase, and therefore, the network may overly fit the seen data. Based on this experiment, we choose 16 as the number of images in a stack, which shows the best FID for the remaining experiments.

We also conducted an ablation study to observe how consistency loss works for feature disentanglement by extracting a common feature from a stacked input. We calculated the L2 distances between the encoded features of different font images from the encoders trained with both stacked input and consistency loss (w $L_{consistency}$) or with only stacked input (wo $L_{consistency}$) and presented the average and standard deviation values for a large number of distance calculations (238 fonts × 302 characters for seen data and 40 fonts × 302 characters for unseen data) in Table 3.

As shown in Table 3, the distance between the encoded features of stacked input is drastically reduced for both text content features (Content) and font style features (Style) when using consistency loss (w $L_{consistency}$) compared to without using consistency loss (wo $L_{consistency}$). This means that the extracted features differ from image to image and that it is difficult to disentangle text content and font style with only the stacked input. However, with consistency loss, a common feature can be extracted from a stacked input (a common character in different font styles or different characters in the same font style) through each encoder and, therefore, disentangled better. These common features from the content stacked input and style stacked input represent text content and font style, respectively.

**Figure 6:**
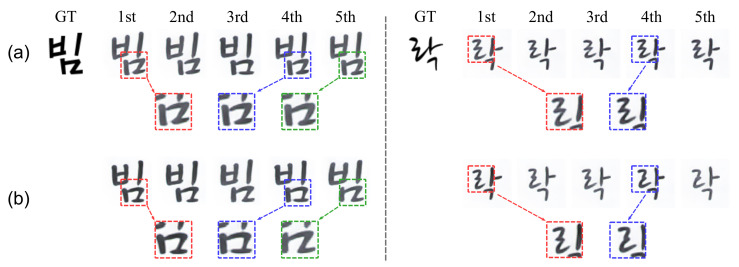
Examples of the generated font images for five trials with random combination of image in the stacked inputs. Each column on both sides of dashed vertical line shows the result with different combinations of stacked input for the same text content and font style: (**a**) w $L_{consistency}$, (**b**) wo $L_{consistency}$.

Each column in the Figure 6 shows some examples of the generated font images from the same unseen font style and text content stacked inputs for five trials with random combinations of images in the stacked inputs. On the left side of the dashed line of the Figure 6, since they are the unseen font generation results, there is a slight difference between the ground truth and the generated image, but the results with consistency loss (Figure 6a) show very similar font style through the five result images. In contrast, when consistency loss is not used (Figure 6b), the generated images are significantly different from each other in the flick of a cursive stroke (red, blue, and green boxes on Figure 6b). Furthermore, on the right side of the dashed line, the results with consistency loss (Figure 6a) are not only relatively more similar to the ground truth but also more consistent than the results without consistency loss (Figure 6b) in the detailed style such as cursive strokes (red and blue boxed on right side of the dashed line in Figure 6). This shows that our consistency loss enables consistent font generation regardless of image combination in the stacked inputs by doing feature disentanglement.

As shown in Table 4, the FID performance is also improved with consistency loss (90.99 in the second row of Unseen FID) compared to without consistency loss (96.71 in the first row of Unseen FID). Based on this experiment, we used consistency loss for the remaining experiments.

### 4.3. Experiment of Hallucinated Stack

In a real-world situation, it may be difficult to use *R* style images and *R* content images for real stacked inputs. To relieve this requirement, we have already proposed to use the hallucinated stack in the test phase. Here, we experimentally analyze how the hallucinated stack works for arbitrary font generation.

**Figure 7:**
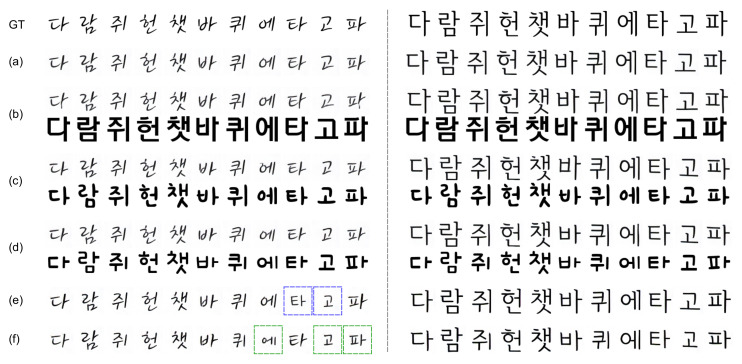
Examples of the generated images for unseen font. GT: image of ground truth font. (**a**): results with real stack, (**b**–**d**): results with hallucinated stack for content reference (upper row) and content images (lower row), (**e**): results with hallucinated stack for style reference, (**f**): results with hallucinated stack for both content and style references.

Figure 7 shows the examples of unseen font generation with or without the hallucinated stack. When we used the hallucinated stack for content reference input and real stack for style reference input, the generated font images (Figure 7b–d) show very similar result regardless of the style of content image and look similar to the results with real stacks for both content and style references (Figure 7a) and the ground truth image (Figure 7 GT). This shows that the content encoder trained by stacked input and consistency loss can extract disentangled content features from a single content image.

However, when we used a real stack for content reference input and a hallucinated stack for style reference input, some of the generated images for the handwritten font (blue boxes on the left side of Figure 7e) look slightly different from the ground truth images. In contrast, for a printed font (right side of Figure 7e), just one image as a hallucinated stack extracted the font style well and resulted in a font generation similar to the ground truth images.

When we used hallucinated stacks for both content and style reference inputs (Figure 7f), similar to previous experiment results (Figure 7b–e), we can find that some results are lack of handwritten stroke in the generated font images (green boxes on Figure 7f). However, while these failures are limited to some specific combinations of consonants and vowels in handwritten fonts, overall experimental results show that our method can generate font images very well with the hallucinated stack of a single image.

### 4.4. Consideration of Residual Blocks

Residual block (ResBlock) [35] has been used to improve performance by making networks deeper. Furthermore, we can add learnability to AdaIN with human-made operations by adding residual blocks on both sides of AdaIN.

**Figure 8:**
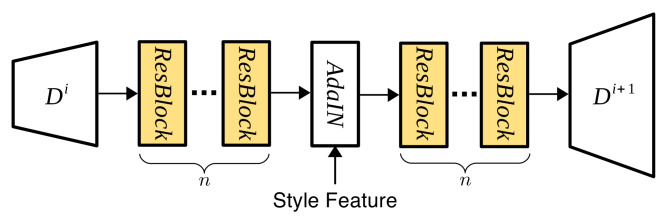
AdaIN with ResBlocks. *n* represents the number of ResBlocks.

To analyze the learnability of AdaIN with ResBlock, we trained networks with a ResBlocks on both sides of AdaIN or without ResBlock as shown in Figure 8.

**Figure 9:**
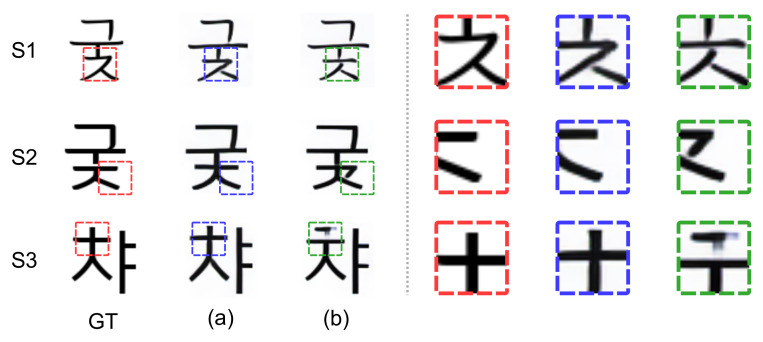
Examples of the generated images for unseen font: GT: image of ground truth font, (**a**): generated images without ResBlock, (**b**): generated images with ResBlock (n=1).

Figure 9 shows some examples of the generated font images with or without a ResBlock. The results with a ResBlock (green box on Figure 9b s2) have a stroke that does not exist in the ground truth image (red box on Figure 9 GT s2). In addition, the positions and shapes of the strokes look different (green box on Figure 9b s1) from the ground truth images (red box on Figure 9 GT s1). An unexpected stroke and a partially erased stroke (green box on Figure 9b s3) also appeared. In contrast, the result without ResBlock (Figure 9a) is similar to the ground truth images without any missing, addition, or deformation of the original strokes.

In Table 4, FID performance is also the best (90.99 in the second row of Unseen FID) without ResBlock for unseen data. For seen data, FID performance with two ResBlocks is the best (67.28 in the fourth row of Seen FID), and this result is the opposite to the previous one for unseen data. These performance results mean that network may overly fit the seen data with ResBlocks and lose general performance for unseen data. Therefore, adding ResBlock to the transformer layer (AdaIN) is not recommended for arbitrary font generation. So, we do not use ResBlock for the remaining experiments.

### 4.5. Style Interpolation

**Figure 10:**
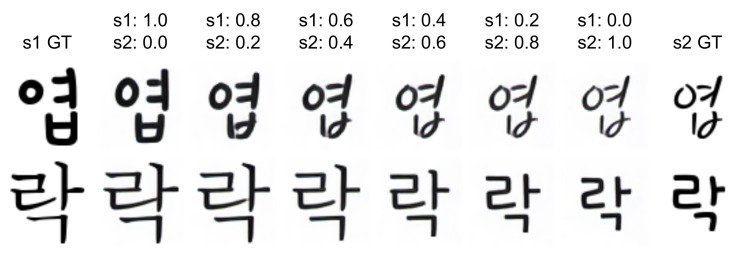
Examples of style interpolation between two unseen font styles. The first row represents interpolation weights for two styles.

In style transfer [18,29], two or more styles can be mixed to make a new style, and this technique is called style interpolation. Our font generation method is compatible with this interpolation technique. To enable interpolation in our method, the operations (Equations (8) and (9)) are applied to all AdaIN layers of the decoder network:

(8) $$I\left(x,s_{1,\cdots ,n},w_{1,\cdots ,n}\right)=\sum_{k=1}^{n}w_{k}\times \mathrm{AdaIN}\left(x,s_{k}\right),$$

(9) $$\sum_{k=1}^{n}w_{k}=1,$$

where $s_{1,\cdots ,n}$ represent the styles for interpolation, I(·) is the interpolated feature and passes through the next layer of the decoder, $w_{k}$ represents the interpolation weight of the *k*-th font style, and *n* represents the number of font styles. To show how well our method works with style interpolation, we conducted an interpolation experiment with real stack inputs as shown Figure 10. As shown in Figure 10, our network can interpolate the two font styles as the background is kept clean. The size of the interpolated font changes smoothly from one font style (the first column in Figure 10) to the other font style (last column in Figure 10). This shows that our network can generate not only one given font style but also an interpolated font style from given multiple styles.

### 4.6. Comparison to the Previous Methods

In this section, we compare our font generation method with real stacks (our-real) or with hallucinated stacks (our-hall) with the previous arbitrary font generation methods [12,13] that use two separate encoders for arbitrary font generation. We used the same training and testing datasets in Section 4.1 for all methods, and for the other settings, we followed the original settings of the papers [12,13]. We omit component-wise encoding approaches [15,20,21] from the comparison group because they need additional labels.

For quantitative evaluation, we adopted two additional evaluation metrics in addition to FID. For pixel-level evaluation, we employ the L1 distance between the generated image and the ground truth image. For human-perceptual-level evaluation, we also consider the perceptual distance (PD) [36] and the L2 distance of features between generated image and ground truth image. We calculated those evaluation metrics after generating 302 characters for each font in the testing dataset in Section 4.1 with all methods. We presented the average and standard deviation of the metric values in Table 5.

The overall performance of Ours-real is the best among the four methods since Ours-real has the lowest metric values for both seen and unseen data. This result shows that Ours-real outperforms the other methods in arbitrary font generation. Although Ours-hall performs worse than Ours-real because it sometimes failed to generate handwriting fonts, as shown in Figure 7f, Ours-hall still performs better in L1 and PD than FUNIT and in FID than EMD for unseen data. However, we cannot conclude that Ours-hall is better than the previous method because the differences are quite small. Therefore, we additionally analyzed the qualitative performances of those methods as a complementary comparison.

**Figure 11:**
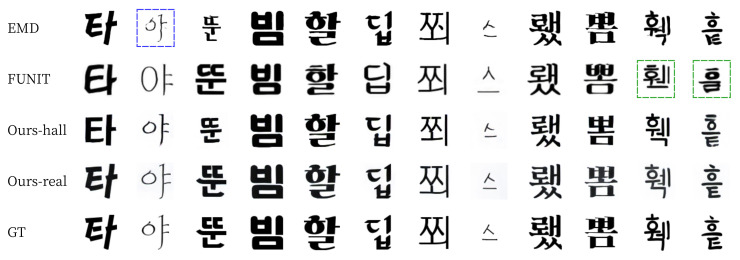
Examples of Korean seen font style generation. The presented characters were not shown in the training phase.

**Figure 12:**
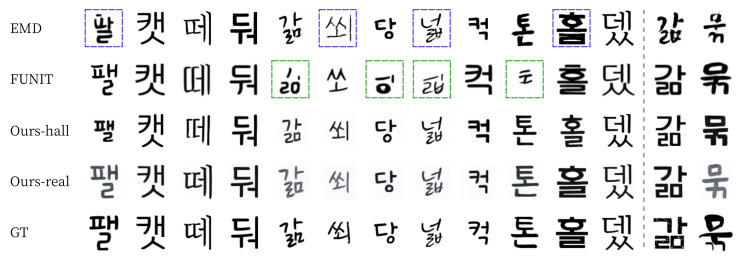
Examples of Korean unseen font style generation. The presented characters were not shown in the training phase. The last two columns show some failure cases.

For qualitative evaluation, we provide visual comparisons in Figure 11 and Figure 12. For seen data, EMD often erased the strokes of the thin letters (blue box on Figure 11) [15]. FUNIT failed to generate fonts with detailed strokes and often broke the structure of the content (green boxes on Figure 11). In contrast, our methods (Ours-hall and Ours-real) generated thin letters and detailed strokes (images on the third and fourth rows of Figure 11) very similar to ground truth images (images on the last row of Figure 11). For unseen data, FUNIT also failed to generate handwriting fonts (green boxes on Figure 12). Similarly, EMD has a problem of unstable handwriting fonts even when thin stroke does not exist (blue boxes on Figure 12). In contrast, our methods (Ours-hall, Ours-real) generated unseen font similar to ground truth images (GT) without the collapse of the structure of glyph. The last two columns of Figure 12 show some failure cases of unseen font generation. The images on the 13th column show failures in generating the pattern of small holes on the strokes of GT. Ours-real could not generate the hole patterns in the output image. However, the shape and scale of the output image generated by Ours-real is the closest to GT while both END and FUNIT generated totally different font styles from this GT font style. The images on the last column show failures in generating the font style of GT with strokes of varying sizes. All methods failed in this case.

The only drawback of our methods is the lighter pixel intensity compared to EMD and FUNIT. This is because the perceptual loss reduces the distance between the gt image and the generated image at the feature level. In this process, the intensity at the pixel level is relatively ignored, resulting in a lighter result. However, this can be easily solved by additional post-processing such as binary thresholding.

**Figure 13:**
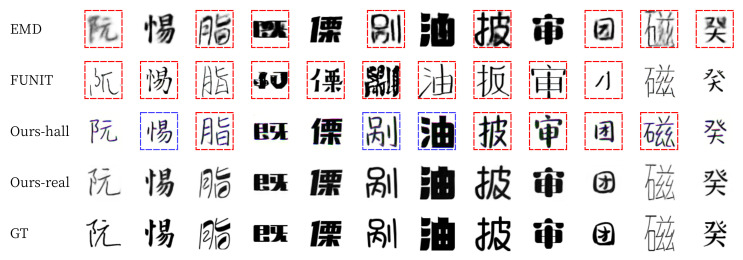
Examples of Chinese seen font style generation. Red boxes represent ‘bad’ results, blue boxes ‘not bad’, and no box ‘good’ result.

**Figure 14:**
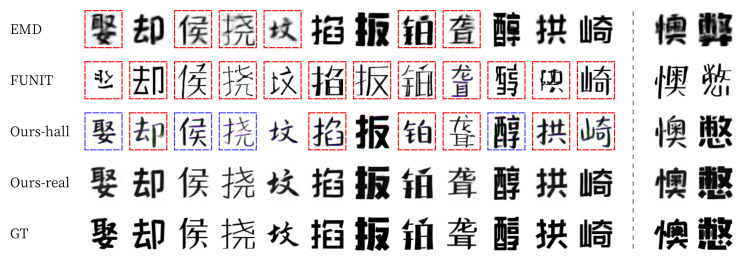
Examples of Chinese unseen font style generation. Red boxes represent ‘bad’ results, blue boxes ‘not bad’ results, and no box ‘good’ results. The last two columns show some failure cases, so we did not mark any box here.

Since our method is not limited to Korean characters, we performed additional experiments of font generation for Chinese and English characters to verify the generalization performance of our methods with different languages. Figure 13 and Figure 14 show the result images for Chinese characters of complex structure, where the images without box are ‘good’ results, with red box are ‘bad’ results, and with blue box are ‘not bad’ results. Ours-real shows the most similar font images to GT in both seen and unseen font generation. Ours-hall, which uses a hallucinated stack of a single input image, shows worse results for Chinese characters (several ‘bad’ and ‘not bad’ images) than for Korean characters because of difficulty in generating more complex characters. In contrast, FUNIT shows many failure output images of distorted character with missing strokes and different font styles from GT in both seen and unseen font generation. EMD also failed to generate many font images and especially thin fonts in both seen and unseen font generation. The last two columns of Figure 14 are two failure cases of our methods for unseen fonts and characters. In these failure cases, Ours-real shows blurry images but font style and character similar to GT. Ours-hall shows a little different font style from GT but more similar to GT than those of FUNIT. EMD shows very blurry characters different from GT.

**Figure 15:**
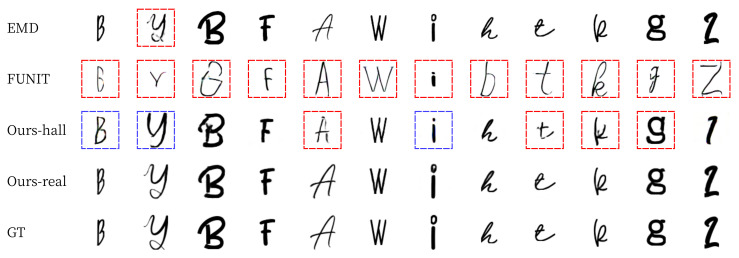
Examples of English seen font style generation. We show the ‘bad’ results with red box, ‘not bad’ result with blue box, and ‘good’ result without box.

**Figure 16:**
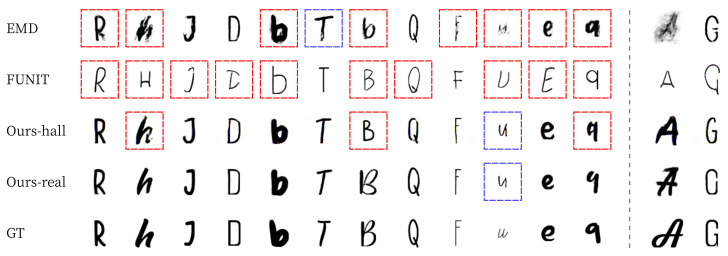
Examples of English unseen font style generation. Red boxes represent ‘bad’ results, blue boxes ‘not bad’ results, and no box ‘good’ results. The last two columns show some failure cases, so we did not mark any box here.

Figure 15 and Figure 16 shows the results of English alphabet font generation, where the images without a box are ‘good’ results, with a red box are ‘bad’ results, and with blue box are ‘not bad’ results. Similar to the results of Korean and Chinese characters, Ours-real shows the best images similar to GT in both seen and unseen font generations. Ours-hall shows worse results than EMD in seen font generation but better results in unseen font generation, which is a more general application than seen font generation. FUNIT shows the worst results in both seen and unseen font generations. The last two columns of Figure 16 show some failure cases of our methods. Even for these failure cases, Ours-real and Ours-hall made font images similar to GT while EMD and FUNIT generated incomplete images or images with different font style from GT.

**Figure 17:**
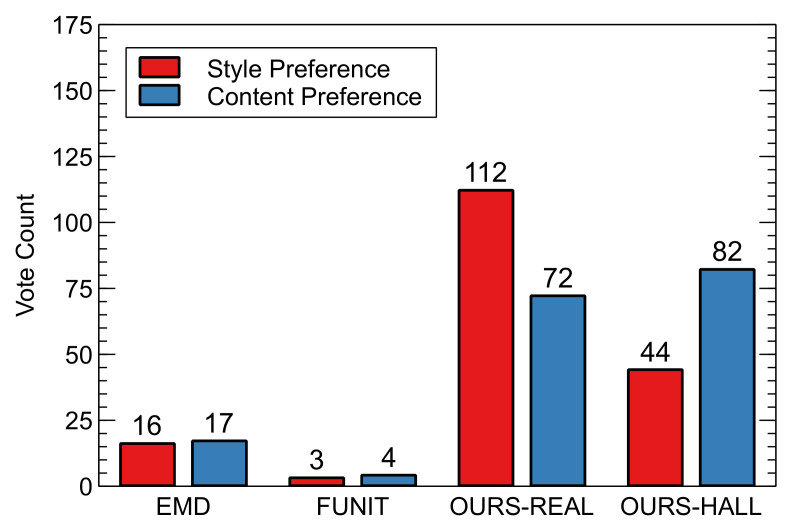
Result of user study. The maximum number of votes for each question is 175.

We conducted a user study for human-level evaluation. We measured style preference, the preference of how well the generated fonts express the style of ground truth, content preference, and the preference of how well the generated fonts express content information for unseen fonts. We showed four images from each method in random order, where those images were randomly selected from Figure 12. Then, for measuring style preference, we asked users to vote for the generated images which are most similar to the presented ground truth font image and, for measuring content preference, we asked users to vote for the generated image that nicely represents the text content of the ground truth character image in different font style from the generated image. After repeating this voting procedure five times, we collected a total of 175 votes from 35 users and presented the total number of votes in Figure 17.

Our methods, i.e., Ours-real and Ours-hall, outperform the other previous methods in both style and content preferences. This means that our methods are preferred in arbitrary font generation. There is no big difference between real stack and hallucinated stack in content preference. However, in style preference, the real stack has a higher preference than the hallucinated stack. This is because it is still difficult for the encoder to extract disentangled style feature from the hallucinated stack of a single style image, and therefore, as shown in Figure 7e, the generated images occasionally fails for handwritten fonts.

## 5. Conclusions

In this paper, we proposed a new network architecture and learning method that can generate arbitrary fonts with a few reference images. We used two separate encoders for content and style feature extraction, respectively, and trained our network with stacked input and consistency loss for common feature extraction. This combination of the separated encoders, stacked input, and consistency loss achieved feature disentanglement and a highly qualified arbitrary font generation performance.

We experimentally proved the effectiveness of our method with ablation studies, quantitative evaluation, and qualitative evaluation. From the ablation study and quantitative evaluation, we found that using our stacked input with consistency loss is effective for two similar images to have similar encoded features and achieved about 11.72 less FID than using a single input for unseen font generation. Compared to the previous methods, our method achieved 17.84 lower FID for unseen data than the previous methods and the user study showed the highest preferences in character and font style generated with our method. In addition, we experimentally confirmed that our method can generate font images using only a single input image (hallucinated stack) in the test phase without much degradation in the generated font image quality compared to using multiple images (real stack).

Moreover, in the experiment of adding ResBlock to AdaIN for the learnable transformer layer, we showed that our method is compatible with additional ResBlock to transformer layer, but using ResBlock is not recommended for a qualified arbitrary font generation result. In the font style interpolation experiment, we also showed that our network collaborating with the previous style interpolation techniques can generate intermediate font styles between two different font styles.

In the perspective of computational cost, our method is not a GAN-based method that needs to train both discriminators and generators, so the computational cost is cheaper than the GAN-based method and easy to train the network. Furthermore, since our method does not use an attention mechanism and only consists of a convolution layer, it is possible to generate fonts in real-time with little memory consumption.

The proposed consistency loss only considers feature sets with the same content or style, i.e., pulling between two features extracted from the same content or style sets, but ignores feature sets with different content or style, i.e., pushing between two features that extracted from different content of style sets. In the future, it is necessary to provide a loss that can consider this relationship, and it seems that contrastive learning can solve this problem.

## Figures and Tables

**Table 1:** Summary of the font generation works. Two encoder means that they split the encoder for content and style. Encoder learning denotes that they proposed the additional loss for the encoder. Component-wise encoding means that they extract the component feature that makes up a character.

	Two Encoder	Encoder Learning	Component-Wise Encoding
EMD [12]	🗸		
W-Net [16]	🗸	🗸	
OCFGNet [17]	🗸	🗸	
SA-VAE [19]	🗸	🗸	
DM-Font [15]			🗸
LF-Font [20]	🗸		🗸
MX-Font [20]	🗸		🗸

**Table 2:** Number (ratio) of training and test fonts for each language.

	Korean	Chinese	Enlish
Train	238 (85%)	185 (92%)	185 (92%)
Test	40 (15%)	15 (8%)	15 (8%)

**Table 3:** Average (and standard deviation) of L2 distances between the encoded features of 16 stacks with or without consistency loss. Bold values indicate the lowest values.

	Seen Data		Unseen Data	
	Content	Style	Content	Style
w $L_{consistency}$	**0.0013**	**0.0035**	**0.0011**	**0.0035**
	(0.0002)	(0.0026)	(0.0001)	(0.0017)
wo $L_{consistency}$	50.1985	0.2347	38.8852	0.2182
	(5.5614)	(0.1684)	(3.7419)	(0.1286)

**Table 4:** Effect of consistency loss and ResBlock on FID. *n* represents the number of ResBlock. Bold values indicate the best performance.

Consistency Loss	ResBlock	Seen FID (↓)	Unseen FID (↓)
×	×	70.05	96.71
∘	×	68.01	**90.99**
∘	∘(n=1)	67.76	101.70
∘	∘(n=2)	**67.28**	102.80

**Table 5:** Quantitative performance comparison. All values are the average (standard deviation) of 302 calculations of each measurement and less is better. The red and blue values represent the best and second best performance respectively.

		L1 (↓)	PD (↓)	FID (↓)
Seen data	Ours-real	**0.082**	**0.059**	**68.01**
		(0.03597)	(0.0381)	(20.84)
	Ours-hall	0.134	**0.117**	99.86
		(0.3741)	(0.0425)	(28.26)
	EMD	**0.116**	0.145	106.86
		(0.0995)	(0.1408)	(60.21)
	FUINT	0.182	0.195	**96.40**
		(0.04778)	(0.0335)	(22.64)
Unseen data	Ours-real	**0.107**	**0.104**	**90.99**
		(0.0503)	(0.0410)	(47.38)
	Ours-hall	0.154	0.131	114.95
		(0.0476)	(0.0405)	(43.01)
	EMD	**0.145**	**0.160**	125.32
		(0.0678)	(0.8663)	(58.79)
	FUINT	0.197	0.210	**108.83**
		(0.04989)	(0.0380)	(44.03)

## Data Availability

Not applicable.
